# Early Basal Leaf Removal at Different Sides of the Canopy Improves the Quality of Aglianico Wine

**DOI:** 10.3390/foods11193140

**Published:** 2022-10-09

**Authors:** Giuseppe Gambacorta, Michele Faccia, Giuseppe Natrella, Mirella Noviello, Gianvito Masi, Luigi Tarricone

**Affiliations:** 1 Department of Soil, Plant and Food Science (DISSPA), University of Bari Aldo Moro, Via Amendola 165/A, 70126 Bari, Italy; 2 Research Centre for Viticulture and Enology, Council for Agricultural Research and Economics (CREA), Via Casamassima 148, 70010 Bari, Italy

**Keywords:** vine defoliation, color intensity, polyphenols, antioxidant activity, anthocyanin profile, volatile profile

## Abstract

It is well known that the early removal of basal leaves is a viticultural practice adopted to improve the exposure of clusters to direct sunlight and UV radiation and increase the phenolic compounds and anthocyanin concentration in the berries. The aim of this work was to study the influence of early basal leaf removal on Aglianico wines produced in the Apulia region (southern Italy) during three consecutive seasons. Three vine treatments were carried out, where 100% of the cluster-zone leaves on the north, south and both sides of the canopy were removed. Undefoliated plants were used as a control. The effect of the treatments on the basic chemical parameters, phenol content and volatile composition of wines was investigated using WineScan FT-MIR, spectrophotometry, HPLC-DAD and SPME-GC/MS. Early defoliation increased the amounts of flavonoids (+35–40%), anthocyanins (+15–18%), total polyphenols (+10%), antioxidant activity (+8–14%) and colour intensity (+10%), especially when leaf removal was applied on the south side. Moreover, leaf removal led to a 40% increase in free anthocyanins when applied on the south side of the canopy, while a 24% increase was observed when applied to the north side and 21% when applied to the north and south sides. A negative effect was observed on volatile compounds, which decreased by about 18, 14 and 13% when the treatment was applied on the north, north-south and south sides of the canopy, respectively. In conclusion, early leaf removal treatments allow for the modulation of the phenolic and volatile concentrations of Aglianico wines.

## 1. Introduction

The microclimate of the vine canopy in terms of sunlight exposure and air temperature and circulation has a considerable effect on the colour of red grapes and consequently the quality of wine. Nowadays, global warming is responsible for climate change, which has exerted strong effects on grapevine phenological phases, such as grape ripening and the relative chemical composition of the berries, reflected in vine yields and wine quality. The increased frequency of periods with high air temperatures negatively affects grapevine water conditions, leading to a decrease in the total acidity, anthocyanin content and aromatic profile of berries at harvest, with negative consequences on the aromatic complexity and freshness of wine [1,2]. Moreover, high air temperatures cause the appearance of sunburn phenomena in leaves and berries [3,4]. The modulation of phenolic content and composition is the main goal of viticultural practices due to its associated effects on the quality of red grapes and wines [5]. For this purpose, several attempts have been made to adopt suitable viticultural practices [6,7], while other studies have explored these practices as an answer to climate change [5,8,9,10]. The improvement of the wine bouquet is also of great importance to viticulturists and winemakers due to its impact on wine quality.

Among viticultural practices, early basal defoliation is frequently applied to reduce the incidence of bunch rot and enhance the quality of the grapes and wine by modulating the microclimate around the bunch [11,12,13]. Early defoliation affects the concentration of soluble solids, phenols and anthocyanins in grapes [11,12] and wines [14]. Its positive effects on grape and wine composition are related to the modification of canopy porosity, leaf/fruit ratio, cluster and berry exposure and skin/berry ratio [14,15]. In a three-year study on defoliation applied to cv Barbera in northwest Italy, it was found that when the climatic conditions were less favorable for grape ripening, the quality of the must and wine increased, whereas no effect was observed in warmer years [16]. Pastore et al. [17] reported that defoliation applied to varieties characterized by different anthocyanin and flavonol profiles (Cabernet Sauvignon, Nero d’Avola, Raboso Piave and Sangiovese) showed a genotype-dependent response [17]. Recently, several studies were performed to assess the impact of basal leaf removal on volatile organic compounds in grapes and wine with controversial results. The existing discrepancies among these studies indicate that the grape variety or clone [18,19], climate conditions [20], grape maturity [21] and the timing and severity of leaf removal [20,22,23] may be responsible for the observed effects of basal defoliation on the aromatic properties of grapes and wine.

Aglianico is a late-season red wine grape variety grown in the Apulia, Campania and Basilicata regions in southern Italy, which are characterized by a warm and arid climate. For this variety grown in the geographical area of Potenza (Basilicata), anthocyanin compounds were shown to be increased due to the late defoliation at veraison with or without 40% cluster thinning [24]. In another study on Aglianico (Castel Campagnano, Campania), a reduction in grape sugars and alcohol in wine was observed as a result of two different defoliation intensities performed near harvest time [25]. In contrast, Tarricone et al. [26] found that defoliation applied before flowering (early defoliation) to the basal zone of the canopy at different sides of Aglianico vines (Corato, Apulia) trained to a vertical shoot position system did not affect the sugar content, pH or total acidity of grapes. Moreover, the authors detected an increase in proanthocyanidins, total polyphenols and antioxidant activity in grapes when leaf removal was applied to the basal shoots on the south side of the canopy.

The goal of this work was to evaluate the impact of manual early basal leaf removal on the basic chemical parameters, polyphenolic fractions, anthocyanin composition and volatile organic compounds of Aglianico wines produced in a Mediterranean environment. For this purpose, three different defoliation treatments were performed on three different sides of the vines during the 2016, 2017 and 2018 seasons.

## 2. Materials and Methods

### 2.1. Vineyard, Leaf Removal Treatment and Vinification

The early leaf removal experiments were carried out in a commercial Aglianico (*Vitis vinifera* L.) vineyard situated in the Apulia region, southern Italy, near Corato, 41°04′35″ N 16°21′46″ E, 354 m a.s.l., during the 2016, 2017 and 2018 seasons, as detailed in our previous study [24]. In the vineyard, six rows of 50 m were selected to set up a randomized complete block design, with two rows as a block separated by a buffer row. Within every two rows, three sections of 18 vines per plot were tagged and randomly assigned to the leaf removal treatment groups, with 54 vines for each treatment. For each treatment, 3 replicates were performed (18 × 3 = 54 vines). The removal of the basal part of the shoot up to the last cluster was manually performed 15 days before flowering. The following early leaf removal treatments were applied:

- Control (C): no leaf removal, where all basal leaves were retained in each shoot;
- Leaf removal on the south canopy side (S): 100% of fruit-zone leaves on each shoot were removed from the south canopy side;
- Leaf removal on the north canopy side (N): 100% of fruit-zone leaves on each shoot were removed from the north canopy side;
- Leaf removal on the north-south canopy side (NS): 100% removal of fruit-zone leaves on both the north and the south side of the canopy.

For each trial, about 100 kg of grapes were manually harvested and vinified at the experimental winery of Bari University according to the procedure described by Coletta et al. [27]. Briefly, the grapes were crushed and de-stemmed with a stainless-steel crusher-destemmer (Enotecnica Pillan, Camisano Vicentino, Italy) and placed in 100 L vertical stainless-steel vats. Potassium metabisulphite (6 g/100 kg), yeast (*Saccharomyces cerevisiae* var. Bayanus, Mycoferm CRU05, 20 g/100 kg, Ever, Pramaggiore, Italy) and yeast activator (Enovit, AEB, Venice, Italy) were added. Maceration was performed for 9 days with 2 punch-downs per day. Then, free-run wine was recovered by draining, and the grape pomace was gently pressed to recover press-run wine using an 80 L stainless-steel hydropress (Enotecnica Pillan, Camisano Vicentino, Italy). The free-run and press-run wines were blended and raked after 2 weeks to eliminate gross lees. The wines were bottled after 6 months, without any treatment, and analyzed. For each wine, three bottles were taken and each was analyzed in triplicate.

### 2.2. Chemical Analysis

An AutoAnalyzer FOSS WineScan FT-MIR spectrometer (FOSS, Padua, Italy) was employed to analyze the chemical characteristics of wines, including ethanol (E, % *v*/*v*), pH, titratable acidity (TA, g/L), malic acid (MA, g/L), lactic acid (LA, g/L), dry reduced extract (DRE, g/L) and ashes (g/L).

### 2.3. Phenols, Colour Indices and Antioxidant Activity

The phenol composition of wines was determined according to the method described by Di Stefano and Cravero [28], while the colour indices (CI, colour intensity and T, tonality) were assessed according to the Glories procedure [29] using a UV–visible spectrophotometer (Beckman Coulter DU 800, Brea, CA, USA). Detailed procedures for the analysis of the flavonoids (F), anthocyanins (A), total polyphenols (TP), proanthocyanidins (P) and flavans reactive with vanillin (FRV) in wines were reported in a previous work [30]. An ABTS assay was used to assess the antioxidant activity of the wines [31], and the results were expressed as mmol/L TEAC (Trolox equivalent antioxidant capacity).

### 2.4. HPLC-DAD Anthocyanin Analysis

The identification and quantification of anthocyanins were performed using a Waters 600 E system including a quaternary pump, Rheodyne injector with a 20 μL loop, column oven and photodiode array detector (Waters, Milford, MA, USA). The separation of anthocyanins was performed using a NovaPack C18 (150 × 3.9 mm, 4 μm, Waters) column set at 30 °C using a flow rate of 1 mL min^−1^. The mobile phase was 10% formic acid (A) and acetonitrile (B). The gradient of solvent A was 0–1 min at 95%, 1–22 min at 60%, 22–23 min at 30%, 23–28 min at 30% and 28–28.1 min at 5%. Anthocyanins were detected at 520 nm, and tentative identification was performed by comparisons with the data reported by Revilla and Ryan [32]. Quantitative analysis was performed according to an external standard method based on a calibration curve obtained by the injection of solutions at different note concentrations of malvidin-3-O-glucoside (R^2^ = 0.9993). The results were expressed as mg of malvidin-3-glucoside equivalents per L of wine.

### 2.5. Volatile Organic Compounds Analysis

The solid-phase micro-extraction (SPME) technique was used to extract volatile organic compounds (VOCs) with a 50/30 μm DVB/Carboxen/PDMS fiber (Supelco, Bellefonte, PA, USA), as described by Filannino et al. [33] with few modifications. Briefly, each sample (1 mL) was inserted into a 20 mL screwcap vial and 0.2 g of NaCl and 10 μL of internal standard solution (2-Octanol, 82 ng) were added. Then, the vials were loaded into a Triplus RSH autosampler (Thermo Fisher Scientific, Rodano, Italy). After sample conditioning at 50 °C for 10 min for equilibration, the fiber was exposed for 15 min to adsorb volatile compounds. Thereafter, the analytes were desorbed in the injector port at 200 °C for 2 min with a splitless time mode of 1 min. The separation of VOCs was performed using a Thermo TRACE 1300 gas chromatograph connected to a Thermo ISQ QD single quadrupole mass spectrometer (Thermo Fisher Scientific, Rodano, Italy) using an Agilent J&W VF-WAX MS capillary column (60 m, 0.25 mm I.D., 0.25 μm film thickness; Agilent, Santa Clara, CA, USA). The chromatographic conditions were as follows: (1) Oven: 40 °C (2 min) to 210 °C at 3 °C/min, held for 3 min. (2) Detector source temperature 250 °C; transfer line temperature 250 °C. (3) Carrier gas: helium at a constant flow of 1.0 mL/min. The electron impact ionization was 70 eV. Data were acquired in full-scan mode in the range of 33 to 200 uma at an acquisition rate of 7.2 Hz. The chromatographic data were acquired using the Xcalibur v2.0 software, while the volatile compounds were identified by comparing the retention times of the experimental peaks with available pure standards and mass spectra with those present in the NIST library. The results were quantified using the internal standard method and were expressed as µg/L of wines.

### 2.6. Statistical Analysis

The OriginPro 2018b software (OriginLab, Northampton, MA, USA) was used to perform the ANOVA and MANOVA analyses to assess the impacts of the season (S), leaf removal (LR) and their interaction. The S and LR interactions of the principal and significant chemical, phenol, anthocyanin and volatile compounds were reported and discussed. All analyses were repeated three times for each sample.

## 3. Results and Discussion

### 3.1. Meteorological Conditions of the Vineyard Site and the Chemical Characteristics of Wines

The vineyard site is characterized by semi-arid conditions, typical of the Mediterranean area. The annual mean temperature is 16.3 °C, where the maximum is reached in August (38.7 °C) and the minimum is observed in February (−2.3 °C); the mean annual rainfall is 563 mm. In the period from April (bud break) to October (harvest), the total rainfall amounts were 445 mm (2016), 128 mm (2017) and 306 mm (2018). In two years (2016 and 2017) the mean of the maximum air temperatures observed during veraison (from July to August) exceeded the annual maximum mean temperature (39.3 °C in 2016 and 41.2 °C in 2017 vs. 38.7 °C); to the contrary, it was lower in 2018 (36.4 °C). The highest accumulated growing degree day was observed in 2017, and the lowest in 2016. In all the three years included in the study, the number of days with maximum temperatures (>35 °C) exceeded the average level of the site. Reference evapotranspiration (ETr) from bud break to harvest was similar in 2016 and 2018, but higher in 2017.

The wines in this study were produced from grapes obtained from an experiment, the results of which were reported in a previous work [26]. Table 1 shows the chemical characteristics of the wines. Season exhibited a strong effect on all parameters (*p* ≤ 0.001). Wines from the 2017 season were the richest in ethanol due to the higher total soluble solids of grapes compared to those from 2016 and 2018 [26]. The wines produced in 2017 also stood out due to their lower total acidity content and for running malolactic fermentation, indicating the full ripeness of the starting grapes. With regard to the impact of leaf removal, it led to an increase in ethanol, pH and DRE, which was more evident in the samples that underwent defoliation at the south and north-south canopy sides. This could be explained by increased ripeness due to increased sun exposure on the south side. Similar results were found in Merlot wine from Mediterranean areas, where the removal of the first 4–5 basal leaves resulted in a higher alcohol content and lower total acidity [34].

**Figure 1:**
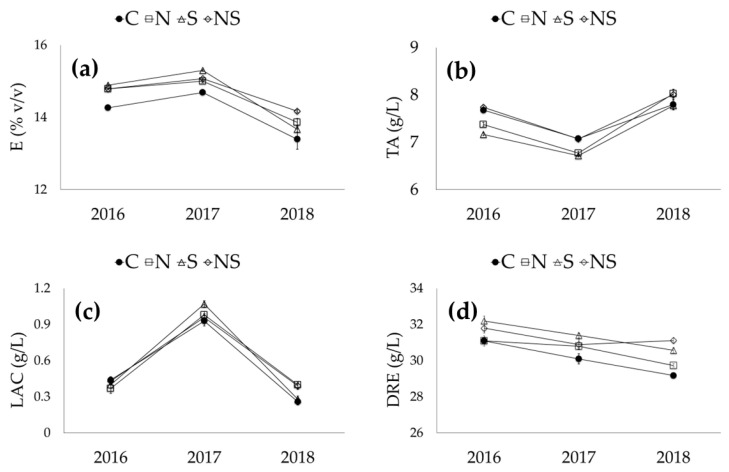
Interaction between season and leaf removal and its effect on the chemical parameters of wines: E—ethanol (**a**); TA—titratable acidity (**b**); LAC—lactic acid (**c**); DRE—dry reduced extract (**d**). C—control; N—leaf removal on the north side of the canopy; S—leaf removal on the south side of the canopy; NS—leaf removal on both sides of the canopy.

Interactions between season and leaf removal (S*LR) were found for E, TA, LAC and DRE, as shown in Figure 1. Defoliation on the south side resulted in an increase in ethanol in 2016 and 2017 and a decrease in 2018 (Figure 1a). Non-defoliated vines produced wines with higher TA in 2016 and 2017; however, the value was lower than that of vines defoliated on the north and north-south canopy sides (Figure 1b). Among the treatments, the wines produced from plants defoliated on the south canopy side presented the highest LAC value in 2017 and the lowest in 2018 (Figure 1c). Finally, defoliation on the north-south sides led to an increase in DRE in 2018 (Figure 1d).

### 3.2. Phenolic Composition

The phenolic composition of the wines, along with their antioxidant activity and color indices, are reported in Table 2. Season exerted a significant effect on all three parameters.

**Figure 2:**
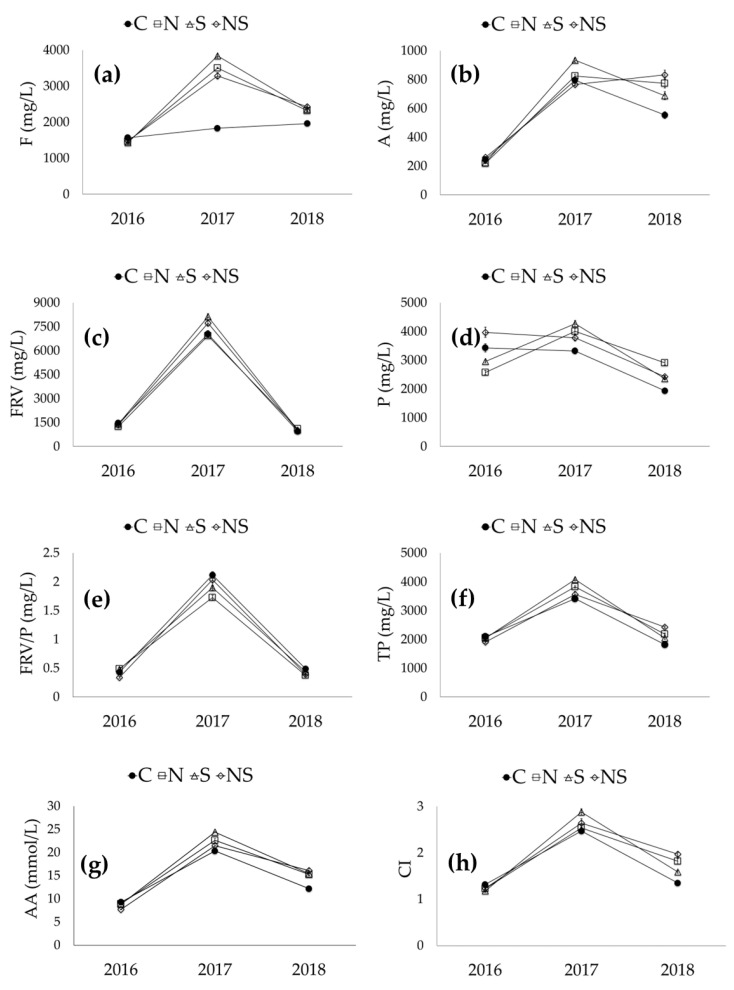
Interaction between season and leaf removal and its effect on the phenolic parameters of wines: F—flavonoids (**a**); A—anthocyanins (**b**); FRV—flavans reactive with vanillin (**c**); P—proanthocyanidins (**d**); FRV/P—flavans reactive with vanillin/proanthocyanidins ratio (**e**); TP—total polyphenols (**f**); AA—antioxidant activity (**g**); CI—color intensity (**h**). C—control; N—leaf removal on the north side of the canopy; S—leaf removal on the south side of the canopy; NS—leaf removal on both sides of the canopy.

As expected, the wines produced in 2017 had the highest contents of phenolic compounds and, consequently, had the highest levels of antioxidant activity and color intensity (*p* ≤ 0.001). This can be explained by the major phenolic maturity of the grapes [26]. In fact, 2017 had the warmest climate and lowest rainfall from bud break to harvest when compared to 2016 and 2018, which induced the best ripening grade of the grapes [26] and, consequently, higher alcohol content, resulting in a major extraction of phenols. When comparing the wines from 2016 and 2018, the latter were characterized by a greater content of anthocyanins, antioxidant activity and color intensity (*p* ≤ 0.001). Early defoliation had a significant impact on the phenolic characteristics, antioxidant activity and color intensity of the wines. In comparison to the control samples, all wines from defoliated vines were richer in flavonoids (+35–40%), anthocyanins (+15–18%), proanthocyanidins (+10–15%), total polyphenols (+10%), antioxidant activity (+8–16%) and color intensity (about +10%). A significant increase in flavonoids, FRV, total polyphenols and antioxidant activity was observed in the wines produced from vines where the leaves were removed from the south side of the canopy, in accordance with the results of other studies conducted on Cabernet-Sauvignon and Probus [35]. As for the FRV/P ratio, which is an index of the degree of tannin condensation, it was found to be lower in the wines produced from defoliated plants, especially those defoliated on the north side (0.87–0.94 versus 1.01 in control). This implies a decrease in tannin reactivity and a predisposition to tannin and color stabilization [36]. Defoliation exerted no influence on tonality. This finding could be explained by the fact that the wines were young, as they were analyzed at an age of about 7 months. Interactions (S*LR) were observed for all phenolic parameters, antioxidant activity and color intensity, as shown in Figure 2.

Compared to the control, leaf removal had no effect on flavonoids in the 2016 wine; in 2018 they were slightly increased and in 2017 they were almost doubled, with a more significant increase when defoliation was carried out on the south side of the canopy (Figure 2a). Defoliation did not influence anthocyanin concentration in 2016, while the highest content was observed in wines from vines defoliated on the south side in 2017 and defoliated on the north and south sides in 2018 (Figure 2b). Evidently, the higher level of defoliation (i.e., on two sides) in 2018 favored greater exposure to the sun and, consequently, greater anthocyanin accumulation. Regarding flavans reactive with vanillin, which potentially contributes to the bitterness of wines [37], leaf removal caused an increase only in 2017 for defoliation performed at the south and north-south sides (Figure 2c). The results concerning proanthocyanidins were much more complex: defoliation on the north side led to lower concentrations in 2016, a value comparable to the other defoliated wines in 2017 and the highest content in 2018 (Figure 2d). Defoliation performed on the north side led to a significant decrease in the FRV/P ratio only in 2017 and, consequently, likely led to the lower bitterness of the wine. (Figure 2e). Defoliation had no effect on total polyphenols in 2016, while it led to an increase in 2017—more significant on the south and north sides—and 2018, where the increase was more significant when carried out on both sides (Figure 2f). As expected, the effect on antioxidant activity was similar to that of total polyphenols: this parameter increased in both the 2017 and 2018 seasons (Figure 2g). Finally, the effect of defoliation on the wine color intensity was similar to that found for anthocyanins: compared to the control, color intensity increased in 2017, especially on the south side, and 2018, with greater prevalence on the north-south and north sides (Figure 2h).

**Figure 3:**
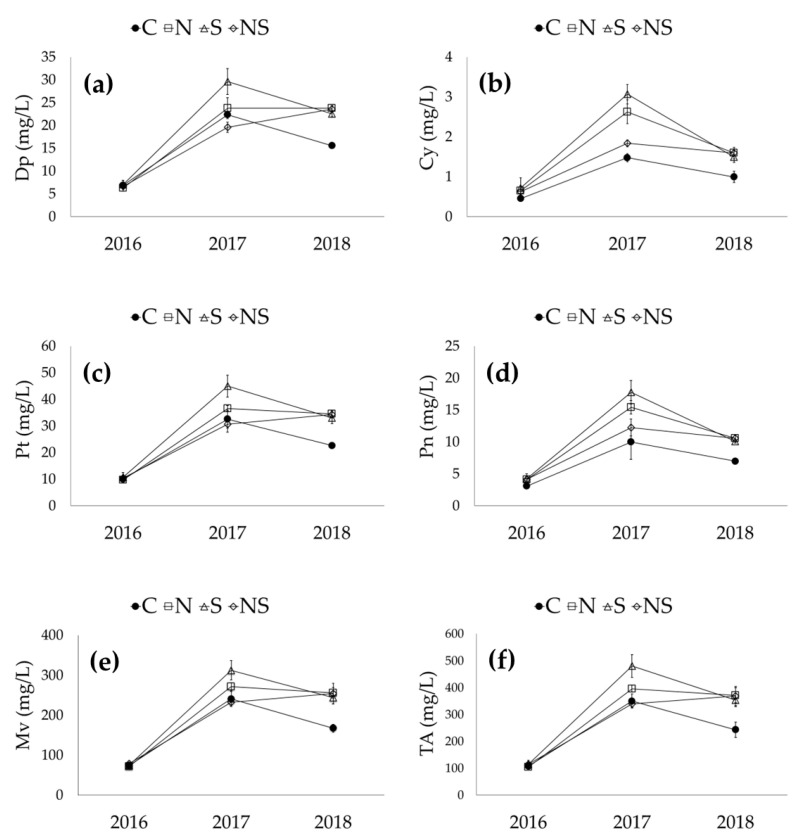
Interaction between season and leaf removal and its effect on the main anthocyanins of wines: Dp—delphinidin-3-glucoside (**a**); Cy—cyanidin-3-glucoside (**b**); Pt—petunidin-3-glucoside (**c**); Pn—peonidin-3-glucoside (**d**); Mv—malvidin-3-glucoside (**e**); TA—total anthocyanins (**f**). C—control; N—leaf removal on the north side of the canopy; S—leaf removal on the south side of the canopy; NS—leaf removal on both sides of the canopy.

### 3.3. Anthocyanins Profile

Table 3 shows the anthocyanin composition of the wines as a function of season and leaf removal. Fourteen anthocyanins were identified and quantified, among which the most abundant were malvidin-3-glucoside, petunidin-3-glucoside, delphinidin-3-glucoside, petunidin-3-glucoside, trans-malvidin-3-coumaroyl-glucoside and peonidin-3-glucoside.

The anthocyanin profile of wines reflects that of the starting grapes; in accordance with the results reported in a previous work and as expected, both season and leaf removal exerted a strong effect on all anthocyanins, which was also observed in the grapes [26]. The 2017 wines were characterized by an increased anthocyanin content, with values 17% higher than those from 2018 and four-fold those from 2016. The wines from the three seasons were characterized by the same anthocyanin composition in terms of the prevalence of non-acylated forms (about 87%) followed by acetylated forms (5.4–5.8%), coumaroylated forms (6.1–6.5%) and the caffeate form of malvidin-3-caffeoyl-glucoside (about 0.7%). Regarding the effect of leaf removal, it caused an increase in anthocyanin content, which was more pronounced when defoliation was carried out on the south side (+40%) with respect to the north (+24%) and north-south sides (+21%). This finding is in agreement with the results reported in a previous work, where defoliation increased the anthocyanin compounds in grapes that were used in winemaking [26]. As expected, the composition of anthocyanins was slightly influenced by defoliation: the proportions of non-acylated forms were 83.4, 85.1 and 88.1% for the south, north-south and north sides, respectively, versus 87.8% in the control wines; acetylated forms accounted for 5.1, 5.2 and 6.4% (north, north-south and south sides, respectively) versus 5.3% in the control; and coumaroyl forms represented 6.5, 9.2 and 9.5% (north, north-south and south sides, respectively) versus 6.2% in the control. Overall, defoliation led to a higher total anthocyanin content in the wines. This finding confirms that the removal of leaves positively affects the synthesis of these compounds. Concerning anthocyanin composition, the treatment caused a slight modification of the profile due to the differing exposure of the fruits to sunlight, UV radiation and temperature [38]. Concerning the S*LR interactions, significant differences were observed for all anthocyanins, with the exception of Pt-Ac.

The S*LR interaction for non-acylated and total anthocyanins is illustrated in Figure 3. With regard to Dp, leaf removal had no effect in 2016, led to an increase on the south side and a decrease on the north-south side in 2017, and resulted in an increase with all three defoliation treatments in 2018 (Figure 3a). Among the defoliation treatments, a decrease in Cy was registered in 2017 (Figure 3b) and a significant increase in Pt was observed with defoliation on the south side in 2017 and on all sides in 2018 (Figure 3c). Regarding Pn, it increased in the 2017 and 2018 seasons; however, this increase was more evident when leaf removal was performed on the south side in 2017 (Figure 3d). Finally, a similar trend to that of Pt was observed for Mv and total anthocyanins: they increased when defoliation was performed on the south side in 2017 and on all sides in 2018 (Figure 3e, f).

### 3.4. Volatile Organic Compounds

Using HS-SPME-GC-MS analysis, a total of 41 volatile compounds were identified and grouped according to the following chemical groups (Table 4): acids (6), alcohols (7), esters (19) and others (9). Briefly, the levels of all VOCs strongly differentiated the season samples (*p* < 0.001). The total VOC content was found to be lower in 2017 and 2018 than in 2016 (about −25% and −49%, respectively). Among the volatile compounds, alcohols comprised the largest group, accounting for 68% of the total in 2016 and 2017 and about 84% in 2018. The second most abundant group was that of esters, which accounted for about 30% in 2016 and 2017 and about 14% in 2018. The other groups were detected at very low levels (1.1–1.4% for acids and <1% for others). It is well known that alcohols are recognizable by their strong and pungent smell and taste [39]. Among this group, 3-methyl-1-butanol (responsible for solvent and fused notes) was the most abundant in all the wines, although the concentrations found were always below its odor threshold (30,000 μg/L) [40]; thus, it likely did not contribute to wine aroma. The second major alcoholic compound was 2-phenylethanol—responsible for honey, spice, rose and lilac notes—found at concentrations above its odor threshold (750 μg/L) [41]; consequently, this compound may have contributed to the aroma of the wines. Concerning esters, diethyl malate, responsible for over-ripe, peach and cut grass odors [42], was the most abundant (148.6–1248.1 μg/L), followed by ethyl octanoate (245.3–775.3 μg/L) with sweet, fruity and pear odors; ethyl acetate (113.1–261.2 μg/L) with pineapple, fruity and solvent odors; ethyl hexanoate (102.6–354.5 μg/L) with fruity, green apple, brandy and wine-like notes; isoamyl acetate (29.1–142.0 μg/L) with banana, fruity and sweet odors; and ethyl decanoate (49.1–116.3 μg/L) with fruity and grape odors [43]. Other esters were present at lower concentrations. Concerning acids, the most abundant was octanoic (42.9–62.6 μg/L), characterized by fatty and rancid notes [43], followed by acetic (9.8–17.6 μg/L) with a pungent and vinegar odor [42], decanoic (9.1–14.0 μg/L) with fatty and rancid notes [43] and hexanoic (2.5–11.7 μg/L) characterized by cheese and fatty notes [43]. However, the concentrations of all these acids were below their odor thresholds and, consequently, they likely did not contribute to the aroma of the wines. Among the other volatiles, sulfur compounds (methionol, ethyl methylthiopropanoate and allyl isothiocyanate), terpenes (α-terpineol and 4-terpineol), 4-ethylphenol, 2-octanone, nonanal and ethyl 2-furoate were identified. All these latter compounds were found at concentrations below their odor thresholds.

The removal of basal leaves influenced the volatile compounds in the wines to a lesser extent than the season. In comparison to the control samples, leaf removal led to a decrease in total volatiles of about 18%, 13% and 14% for defoliation at the north, south and north-south canopy sides, respectively. Concerning the most quantitatively representative group, leaf removal led to a decrease in total alcohols of about 17%, 13% and 11% for north, south and north-south, respectively. A major decrease was observed for 3-methyl-1-butanol and 2-phenylethanol, whereas a slight increase was found for 1-hexanol and benzyl alcohol. A decrease due to leaf removal was also observed for ester compounds and was more pronounced on the north and south sides (−16% for south and −25% for north and north-south, respectively). Almost all ester compounds contributed to this decrease, except for ethyl lactate in the three defoliated treatments, ethyl 2-methylbutyrate and isoamyl acetate in the vines defoliated on the north side, and butyl formate in the vines defoliated on the north and south sides. Defoliation at the south and north-south sides also led to an increase in acids (+21% and +15%, respectively). The greatest increase was observed for octanoic acid; however, the content of 4-ethylphenol (13.5 mg/L versus 20.7–28.3 mg/L) also increased, although the concentrations were about 20 times lower than the odor threshold and thus did not negatively influence the aroma of wines. The same pattern was shown for methionol: it increased with north and north-south defoliation (about double), but at concentrations much lower than its odor threshold.

Overall, this study conducted in three consecutive seasons showed that early leaf removal could induce significant changes in the concentration of volatile compounds in Aglianico wines, which is partially in accordance with other studies [22,44,45]. In fact, the concentrations of the majority of VOCs decreased, while some increased, depending on the side of defoliation: these included 1-hexanol, benzyl alcohol, ethyl 2-methylbutyrate, isoamyl acetate, butyl formate, ethyl lactate, phenethyl acetate, methionol, octanoic acid and 4-ethylphenol (Table 4).

**Figure 4:**
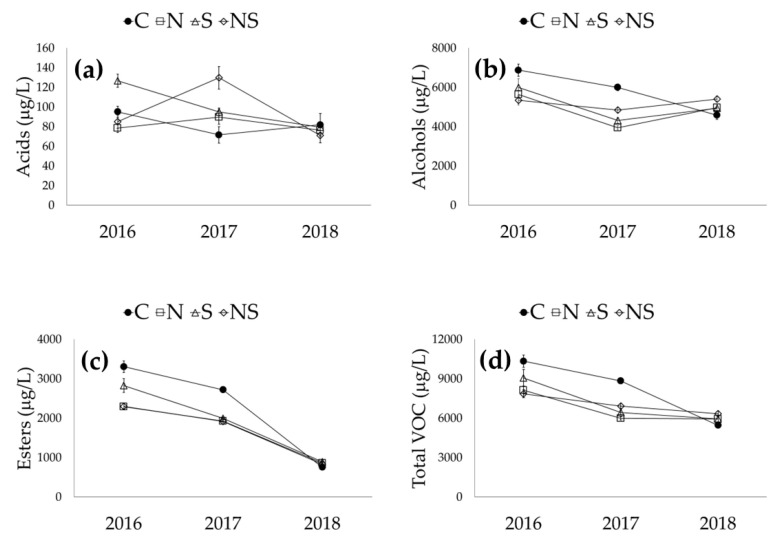
Interaction between season and leaf removal and its effect on the classes of volatile organic compounds in wines. C—control; N—leaf removal on the north side of the canopy; S—leaf removal on the south side of the canopy; NS—leaf removal on both sides of the canopy. (**a**) acids content of wine; (**b**) alcohol content of wine; (**c**) esters content of wine; (**d**) total VOC content of wine.

Regarding the S*LR interactions, significant differences were found for most of the volatiles, except for methyl octanoate, 4-terpineol, ethyl 2-furoate and α-terpineol. The S*LR interactions for total acids, alcohols, esters and total volatiles are illustrated in Figure 4.

As for acids, defoliation caused an increase in 2017, which was more pronounced for defoliation at the north-south sides; an increase was also observed in 2016 but only for the south side, indicating that the impact of defoliation on acids is dependent on both season and the side of defoliation (Figure 4a). The S*LR interaction for alcohols, compounds present at high concentrations in wines, was different. In 2016 and 2017, defoliation caused a decrease in alcohols, while in 2018 an increase was observed (Figure 4b). As for esters, the main volatile compounds produced by yeasts during alcoholic fermentation, defoliation led to a decrease in 2016 and 2017 at the same value as of the non-defoliated vines in 2018 (Figure 4c). Finally, defoliation in the three trials led to a decrease in total VOCs in 2016 and 2017 and a slight increase in 2018; however, these changes were not significant from a statistical point of view (Figure 4d).

## 4. Conclusions

Here, the effect of the basal defoliation of vines on Aglianico wine quality over three consecutive seasons was studied. The obtained results indicated that early basal leaf removal tended to increase the phenolic parameters and decrease the concentration of total volatile compounds in wines, whereas the season, either alone or in interaction with leaf removal, affected all tested parameters. In particular, in the case of high rainfall and a low mean annual temperature, as observed in the 2016 season, the effect on phenols was negligible. Furthermore, leaf removal slightly increased ethanol; this increase was more marked with defoliation at the south and north-south sides, suggesting that the remaining leaf area was sufficient to support grape berry ripening. In conclusion, our data indicate that basal leaf removal before flowering can be used as an effective strategy to increase the total polyphenols, anthocyanins, antioxidant activity and colour intensity of Aglianico wines.

## Figures and Tables

**Table 1:** Chemical characteristics of Aglianico wines as a function of season and leaf removal.

Source of Variation	E (% *v*/*v*)	pH	TA (g/L)	MAL (g/L)	LAC (g/L)	DRE (g/L)	Ashes (g/L)
Season (S)							
2016	^†^ 14.69b	3.33b	7.49b	0.92b	0.41b	31.55a	2.55a
2017	15.02a	3.44a	6.91c	0.10c	0.99a	30.80b	2.51b
2018	13.78c	3.43a	7.91a	1.09a	0.33c	30.16c	2.56a
Significance	***	***	***	***	***	***	***
Leaf removal							
Control	14.12b	3.37c	7.52a	0.72	0.54	30.13c	2.44
North	14.55a	3.39b	7.40b	0.69	0.58	30.55b	2.46
South	14.62a	3.43a	7.22c	0.68	0.58	31.39a	2.51
North-south	14.68a	3.40ab	7.61a	0.73	0.59	31.27a	2.49
Significance	***	**	***	ns	ns	***	ns
Interaction							
S*LR	**	ns	***	ns	***	**	ns

E—ethanol; TA—titratable acidity as tartaric acid; MAL—malic acid; LAC—lactic acid; DRE—dry reduced extract. ^†^ In columns, different letters indicate statistically significant differences at *p* < 0.05. Significance: ns, ** and *** indicate not significant or significant at *p* ≤ 0.01 or *p* ≤ 0.001, respectively.

**Table 2:** Phenolic characteristics of Aglianico wines as a function of season and leaf removal.

Source of Variation	F (mg/L)	A (mg/L)	FRV (mg/L)	P (mg/L)	FRV/P	TP (mg/L)	AA (mmol/L)	CI	T
Season (S)									
2016	^†^ 1488c	238c	1377b	3235b	0.43b	2033b	8.58c	1.24c	0.62a
2017	3116a	831a	7459a	3848a	1.95a	3727a	22.23a	2.63a	0.47b
2018	2270b	713b	1028c	2413c	0.43b	2123b	14.75b	1.68b	0.47b
Significance	***	***	***	***	***	***	***	***	***
Leaf removal (LR)									
Control	1788c	532b	3155b	2898b	1.01a	2448b	13.94c	1.71b	0.52
North	2432ab	607a	3105b	3167ab	0.87b	2692a	15.62ab	1.86a	0.52
South	2546a	616a	3526a	3201ab	0.94ab	2730a	16.22a	1.88a	0.52
North-south	2401b	620a	3367ab	3395a	0.93ab	2640a	15.09b	1.95a	0.51
Significance	***	***	***	***	**	***	***	**	ns
Interaction									
S*LR	***	***	***	***	***	***	***	***	ns

F—flavonoids as (+)-catechin; A—anthocyanins as malvidin-3-glucoside; FRV—flavans reactive with vanillin as (+)-catechin; P—proanthocyanidins as cyanidin chloride; TP—total polyphenols as gallic acid; AA—antioxidant activity as Trolox equivalent antioxidant capacity; CI—color intensity; T—tonality. ^†^ In columns, different letters indicate statistically significant differences at *p* < 0.05. Significance: ns, ** and *** indicate not significant or significant at *p* ≤ 0.01 or *p* ≤ 0.001, respectively.

**Table 3:** Anthocyanin composition of Aglianico wines as a function of season and leaf removal (mg/L as malvidin-3-glucoside).

Source of Variation	Dp	Cy	Pt	Pn	Mv	Dp-Ac	Pt-Ac	Pn-Ac	Mv-Ac	*c*-Mv-Cm	Mv-Cf	Pt-Cm	Pn-Cm	*t*-Mv-Cm	TA
Season (S)															
2016	^†^ 6.8b	0.6c	10.3c	3.9c	75.3b	1.5b	0.3b	1.1c	3.6c	0.2b	0.8c	0.8b	0.4c	5.8c	111.3b
2017	23.9a	2.2a	36.2a	13.9a	264.8a	5.0a	0.9a	4.0a	13.0a	0.8a	3.0a	2.8a	1.5a	20.4a	392.3a
2018	21.4a	1.4b	31.2b	9.6b	230.9a	5.1a	0.8a	2.3b	9.9b	0.3b	2.3b	2.2a	1.0b	17.0b	335.2a
Significance	***	***	***	***	***	***	***	***	***	***	***	***	***	***	***
Leaf removal (LR)															
Control	15.0b	1.0b	21.8c	6.7b	161.4b	3.5ab	0.5b	1.6b	6.9b	0.2b	1.6b	1.5b	0.9ab	11.9b	234.5b
North	18.0ab	1.6ab	27.0ab	10.1a	200.2ab	4.1a	0.7ab	1.8b	8.3b	0.2b	1.8b	1.7ab	1.1a	15.0a	291.6ab
South	19.8a	1.7a	29.6a	10.7a	210.9a	3.4b	0.6ab	4.7a	12.2a	12.2a	3.0a	2.4a	0.8b	15.7a	327.0a
North-south	16.6ab	1.4ab	25.2b	9.0ab	188.9ab	4.2a	0.9a	1.7b	7.8b	7.8b	1.7b	2.2ab	1.0ab	15.0a	283.4ab
Significance	***	**	***	***	***	**	*	***	***	***	***	***	**	***	***
Interaction															
S*LR	***	*	***	**	***	***	ns	***	***	***	***	**	***	***	**

Dp—delphinidin-3-glucoside; Cy—cyanidin-3-glucoside; Pt—petunidin-3-glucoside; Pn—peonidin-3-glucoside; Mv—malvidin-3-glucoside; Dp-Ac—delphinidin-3-acetyl-glucoside; Pt-Ac—petunidin-3-acetyl-glucoside; Pn-Ac—peonidin-3-acetyl-glucoside; Mv-Ac—malvidin-3-acetyl-glucoside; *c*-Mv-Cm—*cis*-malvidin-3-coumaroyl-glucoside; Mv-Cf—malvidin-3-caffeoyl-glucoside; Pt-Cm—petunidin-3-coumaroyl-glucoside; Pn-Cm—petunidin-coumaroyl-glucoside; *t*-Mv-Cm—*trans*-malvidin-3-coumaroyl-glucoside; TA—total anthocyanins. ^†^ In columns, different letters for each source of variation are significantly different by LSD test at *p* = 0.05. Significance: ns, *, ** and *** indicate not significant or significant at *p* ≤ 0.05, *p* ≤ 0.01 or *p* ≤ 0.001, respectively.

**Table 4:** Volatile compounds in Aglianico wines (μg/L) as a function of season and leaf removal.

Compounds	Season (S)				Leaf Removal (LR)					Interaction S*LR
	2016	2017	2018	Sig.	Control	North	South	North-South	Sig.	Sig.
Acids										
Acetic acid	^†^ 16.5a	17.6a	9.8b	***	16.5a	9.2b	15.1a	17.8a	***	***
Isobutyric acid	0b	0b	2.2a	***	0.6	0.7	0.9	0.7	ns	*
Hexanoic acid	10.0b	2.5c	11.7a	***	10.4a	5.6b	9.5a	6.8b	***	***
Octanoic acid	56.7b	62.6a	42.9c	***	43.0c	54.0b	62.6a	56.6ab	***	***
Nonanoic acid	0.0b	0.0b	1.6a	***	0.6b	0.4b	0.2c	0.9a	***	***
Decanoic acid	13.3a	14.0a	9.1b	***	11.9	11.7	12.2	12.6	ns	***
Total acids	96.5a	96.7a	77.3b	***	83.0b	81.6b	100.5a	95.4a	***	***
Alcohols										
2-Methyl-1-propanol	42.5b	41.4b	82.5a	***	53.0bc	50.4c	55.1b	63.3a	***	***
3-Methyl-1-butanol	3063.6a	2853.0b	2991.1a	***	3290.7a	2845.4c	2833.1c	2907.6bc	***	***
3-Methyl-1-pentanol	3.7b	4.7a	2.9c	***	3.8ab	3.8ab	3.5b	4.1a	*	***
1-Hexanol	80.6b	62.9c	105.0a	***	76.3b	83.1ab	82.6ab	89.2a	***	***
2-Ethyl-1-hexanol	2.9a	1.3b	2.6a	***	2.5a	1.8b	2.5a	2.2ab	**	***
Benzyl alcohol	5.7a	0.0c	1.8b	***	0.6b	3.3a	2.8a	3.3a	***	***
2-Phenylethanol	2769.4a	1818.9b	1793.6b	***	2398.9a	1868.8c	2106.1b	2135.4b	***	***
Total alcohols	5968.4a	4782.2b	4979.5b	***	5825.8a	4856.6c	5085.7bc	5205.1b	***	***
Esters										
Ethyl acetate	261.2a	198.2b	113.1c	***	216.2a	190.0b	172.2b	185.0b	***	***
Ethyl propanoate	49.4a	42.9b	2.9c	***	38.3a	27.1b	30.9b	30.7b	***	***
Ethyl isobutyrate	48.0a	40.5b	5.4c	***	40.9a	28.1b	29.3b	26.8b	***	***
Ethyl butyrate	12.4a	12.8a	8.9b	***	12.9a	10.3b	11.1b	11.1b	**	***
Ethyl 2-methylbutyrate	46.9a	7.3b	6.6b	***	27.0b	10.3c	30.9a	12.8c	***	***
Ethyl isovalerate	38.7a	7.2b	4.9c	***	25.7a	10.5c	19.5b	12.0c	***	***
Isoamyl acetate	81.7b	142.0a	29.1c	***	84.6b	78.2b	91.4a	82.9b	***	***
Butyl formate	1.7b	2.0b	2.4a	***	1.7b	1.9b	1.6b	2.9a	***	***
Ethyl hexanoate	206.1b	354.5a	102.6c	***	262.4a	194.6c	229.7b	197.6c	***	***
Hexyl acetate	1.8b	3.6a	1.7b	***	2.6a	2.2b	2.3b	2.3b	*	***
Ethyl heptanoate	2.0a	2.2a	1.6b	***	2.3a	1.5b	2.0a	2.0a	***	*
Ethyl lactate	19.3a	10.4b	6.6c	***	8.4c	19.0a	11.0b	10.0bc	***	***
Methyl octanoate	1.2a	1.2a	0.8b	***	1.2a	0.8b	1.2a	1.1a	***	ns
Ethyl octanoate	484.0b	775.3a	245.3c	***	670.2a	410.1c	548.3b	377.7c	***	***
Ethyl decanoate	86.8b	116.3a	49.1c	***	122.3a	58.9c	93.3b	61.7c	***	***
Diethyl malate	1248.1a	336.4b	148.6c	***	649.1a	562.8b	534.9b	563.9b	**	***
Phenethyl acetate	0.0b	0.0b	23.6a	***	7.2b	6.0c	6.7c	11.6a	***	***
Diethyl succinate	61.8a	31.1b	9.4c	***	39.4a	31.6b	29.8b	35.7b	***	***
Monoethyl succinate	33.3c	58.4b	74.1a	***	51.5	52.1	60.8	56.5	ns	***
Total esters	2684.4a	2142.3b	836.7c	***	2263.9a	1696.0c	1906.9b	1684.3c	***	***
Others										
2-Octanone	4.2a	2.8b	2.1c	***	3.2a	3.2a	3.1a	2.7b	**	*
Allyl isothiocyanate	4.9a	2.5c	2.9b	***	3.2b	2.0c	4.2a	3.3b	***	***
Nonanal	2.3b	3.1a	1.5c	***	2.6	2.4	2.5	2.3	ns	***
Ethyl methylthiopropanoate	8.6a	5.3b	3.2c	***	5.9	6.7	5.5	6.5	ns	***
4-Terpineol	0.0b	2.3a	0.0b	***	0.8	0.8	0.8	0.9	ns	ns
Ethyl 2-furoate	3.6b	3.9a	0.0c	***	1.3	1.0	1.1	1.3	ns	ns
Methionol	26.0a	8.3c	10.8b	***	10.3c	17.8b	11.2c	20.8a	***	***
α-Terpineol	12.7a	3.7b	0.0c	***	5.5	5.2	5.3	4.8	ns	ns
4-Ethylphenol	18.7a	3.1c	8.2b	***	13.5c	21.6b	28.3a	20.7b	***	***
Total others	81.0a	35.0b	28.7c	***	46.3b	60.7a	62.0a	63.3a	***	***
Total amounts	8830.3a	7056.2b	5922.2	***	8219.0a	6694.9c	7155.1b	7048.0c	***	***

^†^ In rows, for each source of variation (season and leaf removal), different letters indicate statistically significant differences at *p* < 0.05. Significance: ns, *, ** and *** indicate not significant or significant at *p* ≤ 0.05, *p* ≤ 0.01 or *p* ≤ 0.001, respectively.

## Data Availability

Data is contained within the article.
